# Repairing effects of glucosamine sulfate in combination with etoricoxib on articular cartilages of patients with knee osteoarthritis

**DOI:** 10.1186/s13018-020-01648-z

**Published:** 2020-04-16

**Authors:** Yong Sun, Changde Wang, Chunzhu Gong

**Affiliations:** Shenzhen Pingle Orthopedic Hospital (Shenzhen Pingshan Traditional Chinese Medicine Hospital), 40 Jintang Street, Luohu District, Shenzhen, 518010 Guangdong Province People’s Republic of China

**Keywords:** Cartilage, Etoricoxib, Glucosamine sulfate, Knee osteoarthritis, Repair

## Abstract

**Purpose:**

To evaluate the repairing effects of glucosamine sulfate combined with etoricoxib on articular cartilages of patients with knee osteoarthritis (KOA).

**Methods:**

A total of 106 KOA patients were randomly divided into control (*n* = 40) and experimental groups (*n* = 66) and treated with etoricoxib alone and glucosamine sulfate plus etoricoxib, respectively. Changes in WOMAC score and clinical efficacy were observed. The synovial fluid was extracted. Bone metabolism indices, growth factors, inflammatory factors, matrix metalloproteinases (MMPs), and NO-induced apoptosis-related factors were measured by ELISA. JNK and Wnt5a mRNA levels were determined using RT-PCR.

**Results:**

After treatment, the total WOMAC scores of both groups significantly declined (*P* < 0.05), being lower in experimental group. The total effective rate of experimental group was higher (*P* < 0.05). BGP and OPG levels rose, especially in experimental group (*P* < 0.05). CTX-II, COMP, and RANKL levels decreased, particularly in experimental group (*P* < 0.05). TGF-β, IGF-1, and FGF-2 levels increased, especially in experimental group (*P* < 0.05). Both groups, particularly experimental group, had decreased levels of IL-1β, IL-17, IL-18, TNF-α, MMP-3, MMP-9, and MMP-13 (*P* < 0.05). JNK and Wnt5a mRNA levels of both groups dropped, which were lower in experimental group (*P* < 0.05). NO and LPO levels reduced, being lower in experimental group. SOD level rose, especially in experimental group (*P* < 0.05).

**Conclusion:**

Glucosamine sulfate plus etoricoxib can repair the articular cartilages of KOA patients. Probably, JNK and Wnt5a are downregulated to inhibit the secretion of MMPs through lowering the levels of inflammatory factors, thereby delaying cartilage matrix degradation. NO-induced chondrocyte apoptosis may be suppressed via the SOD pathway.

## Background

Knee osteoarthritis (KOA) is a chronic disease of retrograde degeneration of articular cartilage and secondary hyperostosis, which frequently occurs in middle-aged and elderly people, being more common in women. In China, OA occurs in about 3% of people, dominated by KOA. X-ray features of KOA are found in about 60% of people aged above 55 years, and its incidence rate is up to 85% in people aged above 65 [1]. With the prolongation of life expectancy and the improvement of quality of life, KOA has aroused increasing attention.

KOA is clinically manifested as joint stiffness, pain, local swelling, deformity, and dysfunction in different degrees. The pathological features of KOA are progressive destruction and degeneration of articular cartilage, subchondral osteosclerosis or cystic changes, joint synovial hyperplasia, osteophyte formation at the joint edge, contracture or hypertrophy of the joint capsule, and contracture or relaxation of ligament [2]. The pathological mechanism of KOA remains unclear, and there still lacks specific therapeutic methods to reduce pain and control disease progression currently. Therefore, selecting drug or non-drug therapy based on the patients’ conditions is the principle of treatment currently.

Glucosamine sulfate, as a natural amino acid monosaccharide, can supplement the cartilage matrix, delay the cartilage degradation, and promote the synthesis of proteoglycan in chondrocytes, which is a nutritional drug for cartilage [3]. Glucosamine sulfate is able to alleviate the symptoms of joint pain, delay and alter the pathological process of KOA, specifically supply the cartilage matrix in articular cartilage, and restore the normal metabolism. Thus, it is the only effective drug that can block the vicious pathological circle of KOA and promote the cartilage repair currently. As a specific inhibitor of cyclooxygenase-2 (COX-2) and a non-steroidal anti-inflammatory drug, etoricoxib has analgesic, anti-inflammatory, and antipyretic effects [4], which has been widely used to relieve pain, reduce morning stiffness, and improve the joint function of OA and rheumatoid arthritis patients. In this study, glucosamine sulfate in combination with etoricoxib were used to treat KOA, and the mechanism for articular cartilage repair was explored, aiming to provide references for clinical KOA treatment.

## Materials and methods

### Subjects

This study was approved by the ethics committee of our hospital (approval no. SPOH201601003), and written informed consent was obtained from all patients. A total of 106 KOA patients treated in our hospital from January 2016 to May 2019 were selected, including 23 males and 83 females aged 48–75 years with the disease courses of 1–5 years. The patients were numbered in accordance with the sequence of hospitalization and randomly divided into a control group (*n* = 40) and an experimental group (*n* = 66). In the control group, there were 9 males and 31 females with a mean age of 62.07 ± 11.32 years. The mean course of disease was 3.59 ± 0.75 months. In terms of the lesion site, there were 18 cases in the left knee and 22 cases in the right knee. In terms of the Kellgren-Lawrence classification, there were 9 cases of grade I, 15 cases of grade II, and 16 cases of grade III. In the experimental group, there were 14 males and 52 females with a mean age of 61.58 ± 10.24 years. The mean course of disease was 3.74 ± 0.89 months. In terms of the lesion site, there were 35 cases in the left knee and 31 cases in the right knee. In terms of the Kellgren-Lawrence classification, there were 16 cases of grade I, 27 cases of grade II, and 23 cases of grade III. The two groups had comparable baseline clinical data (Table 1).

**Table 1:** Baseline clinical data of subjects ($\overline{\mathrm{x}}\pm \mathrm{s}$) [*n* (%)]

Item	Experimental group (*n* = 40)	Control group (*n* = 40)	*t*/*χ*^2^	*P*
Gender (case)			0.024	0.876
Male	14	9		
Female	52	31		
Age (year)	61.58 ± 10.24	62.07 ± 11.32	0.229	0.819
Disease course (year)	3.74 ± 0.89	3.59 ± 0.75	0.891	0.375
Lesion site			0.642	0.423
Left	35	18		
Right	31	22		
Kellgren-Lawrence grade			0.285	0.867
I	16	9		
II	27	15		
III	23	16		

### Related criteria

The diagnostic criteria of the American College of Rheumatology were employed [5]: (1) aged above 40 years, (2) pain in the knee most of the time within 1 month, (3) morning stiffness for shorter than 30 min, (4) friction sound of joint during movement, (5) OA symptoms found in synovial fluid test, and (6) osteophyte formation at the joint edge shown in X-ray image. Those who meet the criteria (1), (2), (3), and (4); or (2) and (6); or (2), (3), (4), and (5) were diagnosed as KOA. KOA was graded based on the X-ray image of knee joint according to the Kellgren-Lawrence diagnostic criteria [6]: Grade 0 (normal), grade I (suspicious narrowing of joint space, possibly with osteophytes), grade II (suspicious narrowing of joint space, with obvious osteophytes), grade III (obvious narrowing and sclerosis of joint space, with moderate osteophyte formation), and grade IV (obvious narrowing, sclerosis and deformity of joint space, with massive osteophyte formation).

Inclusion criteria include (1) patients meeting the above diagnostic criteria, with lesions on single knee; (2) those without an allergic history to glucosamine sulfate or etoricoxib; and (3) those who cooperated willingly in this study. Exclusion criteria include (1) Patients complicated with diseases of the endocrine system, digestive system, blood system, heart, liver, kidney, or tumors; (2) those complicated with congenital limb deformity, skin disease, or acute trauma of local knee; (3) those with rheumatoid arthritis, septic arthritis, traumatic arthritis, gout, ankylosing spondylitis, or other rheumatic diseases; (4) those who used corticoids and non-steroidal anti-inflammatory drugs, or immune enhancers, inhibitors, modulators or other decoctions and Chinese patent medicine, or underwent physical therapy; (5) those with mental disorders or disturbance of consciousness; and (6) those who had missing or incomplete medical data, or quit halfway.

### Treatment methods

For the control group, etoricoxib (Merck Sharp & Dohme (Australia) Pty. Ltd., import drug license H20120130, 60 mg × 5 tablets) was orally taken once a day (60 mg/time) for 6 weeks. For the experimental group, glucosamine sulfate capsules (Rottapharm Ltd., import drug license H20170108, 0.25 g × 20 capsules) were orally taken 3 times a day (0.5 g/time) for 6 weeks based on the treatment of the control group.

### Evaluation of therapeutic effects

The knee function was evaluated before and after treatment using the Western Ontario and McMaster Universities Arthritis Index (WOMAC). The scale consists of 24 items in 3 parts. Each score has 4 grades: 0 point (no difficulty), 1 point (mild), 2 points (moderate), 3 points (severe), and 4 points (extremely severe), and the total score is 96 points. There are 5 items for pain (total score of 20 points), 2 items for joint stiffness (total score of 8 points), and 17 items for joint function (total score of 68 points). A higher score corresponds to severer KOA in patients.

The clinical efficacy was determined as follows. Ineffective: Such clinical symptoms as swelling and pain have no changes after treatment, and the knee function has no improvement. Effective: Knee pain occurs sometimes, there is mild pain in walking, the knee function is basically restored, and it is a little uncomfortable to walk up and down stairs. Markedly effective: There is no knee pain at rest, but sometimes pain in activity, the joint function is obviously restored, and neither work nor life is affected. Clinically controllable: The clinical symptoms (swelling and pain in the knee joint) and signs completely disappear, and the joint function returns to normal. Total effective rate = (clinically controllable + markedly effective + effective) / total cases × 100%.

### Detection indices

After iodophor disinfection, the articular cavity was punctured via the lateral patellar approach in a supine position, and the synovial fluid was extracted and stored in a sterile tube. Bone metabolism indices (bone gamma-carboxy glutamic acid-containing protein (BGP), orthopantomography (OPG), crosslinked c-telopeptide of type II collagen (CTX-II), cartilage oligomeric matrix protein (COMP), and cell nuclear factor κB acceptor activating factor ligand (RANKL)), growth factors (transforming growth factor-β (TGF-β), insulin-like growth factor-1 (IGF-1), and fibroblast growth factor-2 (FGF-2)), inflammatory factors (interleukin-1β (IL-1β), IL-17, IL-18, and tumor necrosis factor-α (TNF-α)), matrix metalloproteinases (matrix metalloproteinase-3 (MMP-3), MMP-9, and MMP-13), and NO-induced apoptosis-related factors (nitric oxide (NO), superoxide dismutase (SOD), and lipid peroxidase (LPO)) were measured by ELISA. Total RNA of the synovial fluid was extracted by the Trizol method and reverse-transcribed. RT-qPCR was carried out to detect the mRNA expression levels of C-Jun N-terminal kinase (JNK) and Wnt family member 5a (Wnt5a). Primer sequences for JNK are FP, 5′-CGGGATCTTCAACTTTAACAT GGAAGTGCTTTCTGTGACTTTAAA-3′ and RP, 5′-CCCAAGCTTACTCCTACTAAAAAGCACTTACTTTTAAAGTC-3′. Primer sequences for Wnt5a are FP, 5′-CACACACTACATCAGTGGCTCAAAG-3′ and RP, 5′-TCCAGCACATGAACGTGTAAACAG-3′. Meanwhile, GAPDH (FP, 5′ CTTTAACATGGAAGTGCGGGA-3′; RP, 5′-CTAAAAAGCACTTACCCCAAGCTATC-3′) was utilized as the internal reference. Each sample was tested three times independently.

### Statistical analysis

All data were statistically analyzed by the SPSS 19.0 software. The numerical data were expressed as [*n* (%)] and subjected to the *χ*^2^ test. The measurement data were represented as mean ± standard deviation ($\overline{\mathrm{x}}\pm \mathrm{s}$). The comparisons between two groups were performed by the independent *t* test, and those at different points were conducted with the paired *t* test. *P* < 0.05 was considered statistically significant.

## Results

### WOMAC scores

The pain, joint stiffness, joint function scores, and total WOMAC score of the two groups significantly declined after treatment compared with those before treatment (*P* < 0.05). After treatment, each score and total WOMAC score of the experimental group were lower than those of the control group (*P* < 0.05) (Table 2).

**Table 2:** WOMAC scores ($\overline{\mathrm{x}}\pm \mathrm{s}$)

Item	Experimental group (*n* = 66)	Control group (*n* = 40)	*t*	*P*
Pain				
Before treatment	17.36 ± 2.59	17.18 ± 2.45	0.354	0.724
After treatment	8.94 ± 0.83^ab^	12.57 ± 1.36^a^	17.086	0.000
Joint stiffness				
Before treatment	7.21 ± 0.68	7.25 ± 0.72	0.287	0.775
After treatment	3.14 ± 0.33^ab^	5.07 ± 0.49^a^	24.224	0.000
Joint function				
Before treatment	57.83 ± 6.07	57.36 ± 5.91	0.390	0.697
After treatment	30.62 ± 2.89^ab^	41.27 ± 4.08^a^	15.699	0.000
Total score				
Before treatment	84.16 ± 10.25	84.32 ± 10.43	0.077	0.938
After treatment	41.83 ± 4.09^ab^	63.57 ± 7.38^a^	19.524	0.000

Compared with before treatment, ^a^*P* < 0.05; compared with control group, ^b^*P* < 0.05. *WOMAC* Western Ontario and McMaster Universities Arthritis Index

### Clinical effective rates

The total effective rate of the experimental group was higher than that of the control group (92.42% vs. 67.50%, *P* < 0.05) (Table 3).

**Table 3:** Clinical effective rates

Item	Experimental group (*n* = 66)	Control group (*n* = 40)	*χ*^2^	*P*
Clinically controllable (case)	43	9		
Markedly effective (case)	9	8		
Effective (case)	9	10		
Ineffective (case)	5	13		
Total effective rate (%)	92.42	67.50	20.770	0.000

### Bone metabolism indices

The levels of BGP and OPG significantly rose after treatment compared with those before treatment in both groups, which were higher in the experimental group than in the control group (*P* < 0.05). The levels of CTX-II, COMP, and RANKL significantly decreased after treatment compared with those before treatment in both groups, which were lower in the experimental group than in the control group (*P* < 0.05) (Table 4).

**Table 4:** Bone metabolism indices ($\overline{\mathrm{x}}\pm \mathrm{s}$)

Item	Experimental group (*n* = 66)	Control group (*n* = 40)	*t*	*P*
BGP (ng/mL)				
Before treatment	3.39 ± 0.58	3.42 ± 0.61	0.253	0.801
After treatment	5.78 ± 1.26^ab^	4.13 ± 0.94^a^	7.157	0.000
OPG (pg/mL)				
Before treatment	118.42 ± 12.37	120.51 ± 12.64	0.836	0.405
After treatment	232.85 ± 24.19^ab^	163.79 ± 17.38^a^	15.747	0.000
CTX-II				
Before treatment	2.36 ± 0.27	2.41 ± 0.29	0.899	0.371
After treatment	0.94 ± 0.08^ab^	1.62 ± 0.18^a^	18.569	0.000
COMP				
Before treatment	4.85 ± 0.62	4.90 ± 0.67	0.390	0.697
After treatment	2.59 ± 0.31^ab^	3.46 ± 0.48^a^	11.345	0.000
RANKL				
Before treatment	84.73 ± 9.69	83.82 ± 9.44	0.473	0.637
After treatment	54.38 ± 5.77^ab^	72.95 ± 7.51^a^	14.307	0.000

Compared with before treatment, ^a^*P* < 0.05; compared with control group, ^b^*P* < 0.05. *BGP* bone gamma-carboxy glutamic acid-containing protein, *COMP* cartilage oligomeric matrix protein, *CTX-II* crosslinked c-telopeptide of type II collagen, *OPG* orthopantomography, *RANKL* cell nuclear factor κB acceptor activating factor ligand

### Growth factors

The levels of TGF-β, IGF-1, and FGF-2 were significantly higher in both groups after treatment than those before treatment, being higher in the experimental group (*P* < 0.05) (Table 5).

**Table 5:** Growth factors ($\overline{\mathrm{x}}\pm \mathrm{s}$)

Item	Experimental group (*n* = 66)	Control group (*n* = 40)	*t*	*P*
TGF-β (μg/L)				
Before treatment	22.07 ± 2.16	21.93 ± 2.08	0.328	0.744
After treatment	30.15 ± 3.04^ab^	25.42 ± 2.47^a^	8.313	0.000
IGF-1 (μg/L)				
Before treatment	83.41 ± 10.29	82.87 ± 9.83	0.266	0.791
After treatment	95.72 ± 12.06^ab^	89.64 ± 11.25^a^	2.579	0.011
FGF-2 (ng/L)				
Before treatment	23.58 ± 2.47	24.01 ± 2.53	0.861	0.391
After treatment	34.64 ± 3.59^ab^	28.39 ± 2.92^a^	9.298	0.000

Compared with before treatment, ^a^*P* < 0.05; compared with control group, ^b^*P* < 0.05. *FGF-2* fibroblast growth factor-2, *IGF-1* insulin-like growth factor-1, *TGF-β* transforming growth factor-β

### Inflammatory factors and MMPs

Both groups had significantly decreased levels of IL-1β, IL-17, IL-18, TNF-α, MMP-3, MMP-9, and MMP-13 after treatment compared with those before treatment, being lower in the experimental group (*P* < 0.05) (Table 6).

**Table 6:** Inflammatory factors and MMPs ($\overline{\mathrm{x}}\pm \mathrm{s}$)

Item	Experimental group (*n* = 66)	Control group (*n* = 40)	*t*	*P*
IL-1β (pg/mL)				
Before treatment	74.37 ± 6.95	75.02 ± 7.14	0.462	0.645
After treatment	38.56 ± 3.74^ab^	51.48 ± 4.89^a^	15.322	0.000
IL-17 (μg/L)				
Before treatment	391.64 ± 40.25	389.73 ± 39.52	0.238	0.812
After treatment	205.38 ± 19.76^ab^	276.41 ± 26.11^a^	15.858	0.000
IL-18 (pg/mL)				
Before treatment	232.59 ± 24.16	241.25 ± 23.96	1.794	0.076
After treatment	148.73 ± 13.25^ab^	184.67 ± 17.13^a^	12.099	0.000
TNF-α (pg/mL)				
Before treatment	87.94 ± 9.27	88.31 ± 9.56	0.197	0.844
After treatment	30.52 ± 2.86^ab^	52.45 ± 5.02^a^	28.679	0.000
MMP-3 (ng/ml)				
Before treatment	217.93 ± 20.54	221.01 ± 21.18	0.739	0.461
After treatment	98.46 ± 9.75^ab^	158.37 ± 14.82^a^	21.359	0.000
MMP-9 (ng/ml)				
Before treatment	66.83 ± 7.14	67.25 ± 7.21	0.292	0.770
After treatment	30.26 ± 2.97^ab^	45.38 ± 4.62^a^	20.524	0.000
MMP-13 (ng/ml)				
Before treatment	275.18 ± 28.09	274.92 ± 27.83	0.046	0.963
After treatment	152.43 ± 14.72^ab^	193.76 ± 18.69^a^	10.987	0.000

Compared with before treatment, ^a^*P* < 0.05; compared with control group, ^b^*P* < 0.05. *IL* interleukin, *MMP* matrix metalloproteinase, *TNF-α* tumor necrosis factor-α

### JNK and Wnt5a mRNA levels

**Fig. 1:**
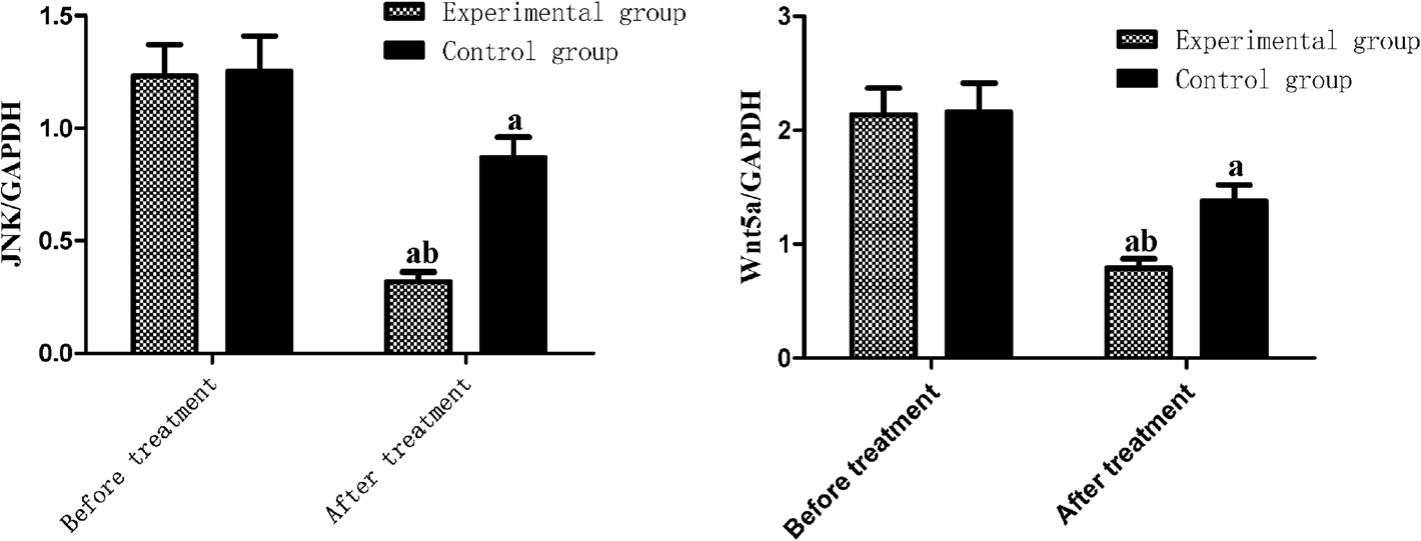
JNK and Wnt5a mRNA levels. Compared with before treatment, ^a^*P* < 0.05; compared with control group, ^b^*P* < 0.05. JNK, C-Jun N-terminal kinase; Wnt5a, Wnt family member 5a

There were lower mRNA levels of JNK and Wnt5a in both groups after treatment than those before treatment, which were significantly lower in the experimental group than in the control group (*P* < 0.05) (Fig. 1).

### NO-induced apoptosis-related factors

Compared with before treatment, the levels of NO and LPO reduced after treatment, which were lower in the experimental group. The level of SOD rose after treatment, being higher in the experimental group (*P* < 0.05) (Table 7).

**Table 7:** NO-induced apoptosis-related factors ($\overline{\mathrm{x}}\pm \mathrm{s}$)

Item	Experimental group (*n* = 66)	Control group (*n* = 40)	*t*	*P*
NO (μmol/L)				
Before treatment	65.82 ± 7.14	66.03 ± 7.21	0.146	0.884
After treatment	27.54 ± 2.69^ab^	42.39 ± 4.28^a^	21.957	0.000
SOD (U/mL)				
Before treatment	69.41 ± 6.87	70.12 ± 6.93	0.514	0.608
After treatment	118.59 ± 12.16^ab^	89.48 ± 9.04^a^	13.096	0.000
LPO (nmol/mL)				
Before treatment	8.74 ± 0.83	8.81 ± 0.86	0.415	0.679
After treatment	1.06 ± 0.15^ab^	3.69 ± 0.42^a^	46.343	0.000

Compared with before treatment, ^a^*P* < 0.05; compared with control group, ^b^*P* < 0.05. *LPO* lipid peroxidase, *NO* nitric oxide, *SOD* superoxide dismutase

## Discussion

KOA is a chronic injury of knee joint cartilage caused by factors such as long-term load bearing and mechanical damage [7]. With the increase of age, the degradation of fibrous tissue, osteoporosis, and changes of synovial fluid composition become more serious resulting in the deficiency of joint cartilage nutrition, the reduction of cartilage lubrication, and the increase of pressure on the bone and articular surface for chronic strain and progressive damage [8]. Meanwhile, osteoproliferation causes osteophytes at the edge of the joint, leading to increased bone pressure, which in turn affects blood circulation, aggravates the degree of injury of articular cartilage, and finally causes symptoms such as swelling, pain, and even dysfunction. Deformation and loss of articular cartilage, regeneration of subchondral bone, and marginal bone are important features of KOA, and articular cartilage is the initial site of the disease. Therefore, in the course of treatment, it is necessary to promote the repair of cartilage tissue while ensuring pain relief and disease progression under control [9]. Glucosamine in human articular cartilage is a basic substance needed in the synthesis of aminoglycans. Oral glucosamine sulfate can directly supplement the cartilage matrix, slow down cartilage degradation, facilitate the synthesis of cartilage protein and the recovery of chondrocyte matrix secretion, and in turn improve the articular cartilage structure [10]. Etoricoxib can selectively inhibit the synthesis of cyclooxygenase and prostaglandin and play an anti-inflammatory role, so as to effectively alleviate joint swelling and pain [11]. The results of this study showed that compared with before treatment, pain, joint stiffness, joint function scores, and total scores of the WOMAC scale were significantly reduced in the two groups after treatment (*P* < 0.05), indicating that both glucosamine sulfate combined with etoricoxib and etoricoxib alone had significant effects. After treatment, the scores of the experimental group and the total score of WOMAC were lower than those of the control group (*P* < 0.05), suggesting that the efficacy of glucosamine sulfate combined with etoricoxib was better than that of etoricoxib alone. The total effective rate of the experimental group was significantly higher than that of the control group (*P* < 0.05), which was consistent with the above results.

BGP, which is secreted by osteoblasts and mainly deposited in bone matrix, is a kind of non-specific collagen that promotes bone mineralization. It is released in large quantities when bone matrix is degraded, which can reflect the activity of osteoblasts [12]. OPG/RANKL/RANK plays a regulatory role in the process of bone formation and absorption [13]. OPG can inhibit the differentiation and maturation of osteoclasts. The maturation and proliferation of RANK and its ligand osteoclasts promote the maturation and proliferation of osteoclasts. The increase in the RANKL/OPG ratio indicates that the imbalance and abnormality of bone reconstruction can also lead to bone loss and bone density reduction [14]. CTX-II, a small polypeptide mainly distributed in cartilage, can be used to reflect the degradation of collagen type II by protease in cartilage. With rising level of CTX-II, the joint damage was aggravated [15]. As a sensitive marker of articular cartilage injury, COMP, which is mainly expressed in cartilage, has high tissue specificity, and its level is positively correlated with the degree of joint injury [16]. Herein, compared with before treatment, BGP and OPG were increased significantly after treatment, and those of the experimental group were significantly higher than those of the control group (*P* < 0.05). CTX-II, COMP, and RANKL were significantly reduced, and those of the experimental group were significantly lower than those of the control group (*P* < 0.05). Thus, glucosamine sulfate combined with etoricoxib in the treatment of KOA can better regulate the bone metabolism indexes of patients, delay or hinder joint degeneration, and improve joint function.

TGF-β is a crucial cartilage repair factor, which can maintain the normal structure and function of articular cartilage. The higher the level of TGF-β is, the more favorable the repair of knee osteoarticular injury will be [17]. IGF-1 can accelerate the proliferation of chondrocytes and the formation of chondrocyte colonies by stimulating chondrocytes to synthesize protein polysaccharides and collagen type II. The expression level of IGF-1 is positively correlated with the response of cartilage repair [18]. FGF-2, an effective angiogenic factor in vivo, can promote the generation of new blood vessels by inducing vascular endothelial cells. In addition, as a mitogen and morphogenetic factor of chondrocytes, FGF-2 can promote the repair of cartilage and bone tissue [19]. This study found that compared with before treatment, the levels of TGF-β, IGF-1, and FGF-2 in the two groups were significantly higher than those in the control group after treatment, and those of the experimental group were significantly higher than those of the control group (*P* < 0.05). Accordingly, glucosamine sulfate and etoricoxib can repair the cartilage tissue of KOA patients, and the combination of the two drugs has a better repair effect.

Inflammatory response is the main pathological feature of KOA; inflammatory factors are important factors that damage joint cartilage and cause swelling and pain, and inflammatory factors mainly play a role through a series of cascade amplification reactions [20]. IL-1β can promote the secretion of MMPs, which leads to the degradation of cartilage matrix and chondrocyte apoptosis. IL-17 exerts a strong induction effect on the catabolism of chondrocytes, thus promoting cartilage degradation, and in addition, it can stimulate the production of IL-6 and indirectly promote the secretion of MMPs [21]. IL-18 can induce the production of nitric oxide and prostaglandin E2, and then participate in the process of KOA inflammation and joint injury [22]. TNF-α can activate and aggregate leukocytes, inhibit the synthesis of type II collagen and proteoglycan, inhibit the self-repair of cartilage, and promote cartilage degradation. MMPs can degrade extracellular matrix of articular cartilage [23, 24]. MMP-3 secreted by synovial cells and chondrocytes can not only degrade matrix protein substrates in a variety of extracellular matrix but also activate enzymes such as MMP-9 and MMP-13 to produce a cascade amplification reaction. MMP-9 can degrade collagen by destroying the reticular structure formed by cartilage matrix and collagen. The synergistic effect of MMP-3 and MMP-9 can accelerate the destruction process and make the changes of collagen and cartilage irreversible. At present, MMP-13, the most effective type II collagen degrading enzyme, can degrade all kinds of collagen and directly damage the integrity of articular cartilage. This study showed that after treatment, the levels of IL-1β, IL-17, IL-18, TNF-α, MMP-3, MMP-9, and MMP-13 in the two groups were significantly lower than those before treatment (*P* < 0.05). After treatment, the levels in the experimental group were lower than those in the control group (*P* < 0.05). Hence, glucosamine sulfate and etoricoxib can inhibit the secretion of inflammatory factors and then reduce the levels of MMPs, so as to slow down the degradation of cartilage matrix and facilitating the repair of damaged chondrocytes. In the process of arthritis cartilage destruction, IL-1β can upregulate the expression of Wnt5a which can upregulate the expression of matrix metalloproteinase through the mediation of JNK signaling pathway [25]. The mRNA levels of JNK and Wnt5a were detected in this study, which were lower after treatment than those before treatment, and those of the experimental group were lower than those of the control group (*P* < 0.05). The results indicated that JNK and Wnt5a were involved in the process of decreasing IL-1β and inhibiting matrix metalloproteinase in the treatment of KOA with glucosamine sulfate and etoricoxib.

As an important pathway leading to chondrocyte apoptosis, NO-induced apoptosis is closely related to NO, LPO, and SOD [26]. A large number of free radicals produced by the body during the onset of KOA can promote apoptosis [27]. NO, a key member of free radicals, can aggravate chondrocyte injury by inducing chondrocyte apoptosis. LPO, the metabolite of free radical lipid peroxidation, can damage the structure of cells and cell membranes. This indicator is often used to indirectly reflect the damage degree of free radicals to tissue cells. SOD can remove oxygen free radicals through disproportionation and block the cytochrome C-dependent mitochondrial apoptosis pathway, thereby playing a protective role in the synovium and chondrocytes. In this study, compared with before treatment, NO and LPO levels were decreased after treatment, and those of the experimental group were lower than those of the control group. Moreover, the SOD level was increased and that of the experimental group was higher than that of the control group (*P* < 0.05). We postulated that glucosamine sulfate combined with etoricoxib dominated in the repair of KOA cartilage by increasing SOD level, inhibiting lipid peroxidation, reducing free radicals, inhibiting apoptosis of chondrocytes, and ultimately protecting and repairing cartilage.

## Conclusions

In summary, glucosamine sulfate combined with etoricoxib may reduce the expression of JNK and Wnt5a to inhibit the secretion of matrix metalloproteinase, and then slow down the degradation of cartilage matrix. It may also inhibit the NO-induced chondrocyte apoptosis through SOD pathway. In the future, we will build animal models or study at the cellular level to further verify this conclusion.

## Data Availability

The datasets used and/or analyzed during the current study are available either online or from the corresponding author on reasonable request.

## Abbreviations

BGP: Bone gamma-carboxy glutamic acid-containing protein
COMP: Cartilage oligomeric matrix protein
COX-2: Cyclooxygenase-2
CTX-II: Crosslinked c-telopeptide of type II collagen
FGF-2: Fibroblast growth factor-2
IGF-1: Insulin-like growth factor-1
IL: Interleukin
JNL: C-Jun N-terminal kinase
KOA: Knee osteoarthritis
LPO: Lipid peroxidase
MMP: Matrix metalloproteinase
NO: Nitric oxide
OA: Osteoarthritis
OPG: Orthopantomography
RANKL: Cell nuclear factor κB acceptor activating factor ligand
SOD: Superoxide dismutase
TGF-β: Transforming growth factor-β
TNF-α: Tumor necrosis factor-α
Wnt5a: Wnt family member 5a
WOMAC: Western Ontario and McMaster Universities Arthritis Index
